# Salicylic Acid for Preserving

**Published:** 1893-12-09

**Authors:** 


### SALICYLIC ACID FOR PRESERVING.

Dr. Thomas Dutton in "The Table" has called attention to the fact that people who eat fruit or vegetables preserved in salicylic acid are taking small doses of salicine daily, and as many persons are extremely sensitive to all salicine preparations, and cannot take the smallest medicinal dose, the effect may be often injurious and even in some few cases fatal. It seems to be extensively used on account of its powerful antiseptic properties to keep certain malt liquors, cider and artificial drinks, as well as many varieties of tinned vegetables. The test for it is simple. Add a small quantity of the solution of chloride of iron (ferric chloride) to the substance suspected to contain salicylic acid. If it be present the mixture will develop a deep violet-red colour. In some articles it is used so freely that its presence may be detected by its pungent biting impression upon the tongue and throat.

